# Design and Parameter Study of Integrated Microfluidic Platform for CTC Isolation and Enquiry; A Numerical Approach

**DOI:** 10.3390/bios8020056

**Published:** 2018-06-18

**Authors:** Amir Shamloo, Saba Ahmad, Maede Momeni

**Affiliations:** School of Mechanical Engineering, Sharif University of Technology, Azadi Ave., 11155-9567 Tehran, Iran; zephyr.ahmad@gmail.com (S.A.); momeni.m@aol.com (M.M.)

**Keywords:** microfluidic biochip, circulating tumor cell, magnetophoretic separation, electroosmotic mixing, numerical simulation, geometry optimization

## Abstract

Being the second cause of mortality across the globe, there is now a persistent effort to establish new cancer medication and therapies. Any accomplishment in treating cancers entails the existence of accurate identification systems empowering the early diagnosis. Recent studies indicate CTCs’ potential in cancer prognosis as well as therapy monitoring. The chief shortcoming with CTCs is that they are exceedingly rare cells in their clinically relevant concentration. Here, we simulated a microfluidic construct devised for immunomagnetic separation of the particles of interest from the background cells. This separation unit is integrated with a mixer subunit. The mixer is envisioned for mixing the CTC enriched stream with lysis buffer to extract the biological material of the cell. Some modification was proposed on mixing geometry improving the efficacy of the functional unit. A valuation of engaged forces was made and some forces were neglected due to their order of magnitude. The position of the magnet was also optimized by doing parametric study. For the mixer unit, the effect of applied voltage and frequency on mixing index was studied to find the optimal voltage and frequency which provides better mixing. Above-mentioned studies were done on isolated units and the effect of each functional unit on the other is not studied. As the final step, an integrated microfluidic platform composed of both functional subunits was simulated simultaneously. To ensure the independence of results from the grid, grid studies were also performed. The studies carried out on the construct reveal its potential for diagnostic application.

## 1. Introduction

Exhibiting a growing trend, cancer is now the second cause of mortality worldwide. According to GLOBOCAN statistics, just in 2012, about 14.1 million new cancer cases had been diagnosed and 8.2 million deaths had been documented [1]. It has been identified that most cancer patients would survive if their disease had been diagnosed at early stages. CTCs, which are shed from primary tumor in to blood stream, have provided new tools for cancer diagnosis [2,3,4]. The current technology for isolation of these rare cells from blood stream, namely FACS and MACS, do not provide the supreme technique as they are labor intensive, time consuming and require an adroit operator. 

Upon emergence of microfluidic technology, they are now considered as alternative to conventional separation methods. As it can be alluded by their names, they work in micron sizes and more precise control can be exerted on processes. In addition, their small footprint allows for portability. They also eliminate the perquisite of a skillful operator [5,6,7,8].

In a microchannel, the separation can be achieved both by active and passive techniques. In passive technologies, the main focus is on channel geometry and the cells are distinguished by means of their hydrodynamic differences such as size, density or deformability [9,10]. The filtration technique, for instance, is a passive separation technique which splits cells based on variations in both size and deformability. A substantial challenge with this method is that, in most cases, it induces cell damage and irreversible change in cytoskeleton. Besides, clogging and saturation of the filter are other problems that result in irregular flows and loss of the filtration efficiency. Active technology, on the other hand, relies on application of external force. Active separation techniques generally allow for more precise control over target particles. Additionally, active methods are tunable in real time, they yield more efficient isolation and the channel possesses a simpler geometry. Optical separation, acoustophoresis, dielectrophoresis, and magnetophoresis can be stated as major routes for active separation [9].

Each of the active separation techniques has its pros and cons. For instance, in optical separation, heating from laser beam can potentially denature biological entities and complex underlying theory of acoustophoresis renders it rather unappealing for cell isolation. Dielectrophoresis solely relies on dielectrophoretic force (DEP), which arises from interactions of a cell’s induced dipole and its surrounding spatial gradient of electric field. While it provides high throughput, performance strictly depends on the electrical properties of the liquid medium, particle shape, and its effective dielectric constant. In addition, the alternating electric field may polarize the cell membranes and lead to cell death [11]. On the contrary, magnetophoretic separation, which employs magnetic fields for isolation, has attracted a growing interest owing to its simplicity and non-invasiveness. This method is free of induced heat in most cases and independent of solution pH and ionic concentration. Magnetic force is not directly in contact with cells, minimizing potential hazardous effects that can reduce the viability of cells, which typically associated with dielectrophoresis. This technology can be mainly categorized as positive and negative magnetophoretic methods. In “positive Magnetophoresis” magnetic beads are used to manipulate cells. It involves labeling cells of interest with magnetic beads. Because the magnetization of beads is larger than its surrounding medium, cell-bead conjugates are magnetized under external fields and therefore move towards the location of field maxima [12].

Haung et al. [13] devised a microfluidic chamber for CTC isolation based on immune-magnetic separation principles. The polydimethylsiloxane channel was fabricated by standard molding procedure and bonded to a glass coverslip to form the chamber through which the blood flows. Then, the microfluidic chamber was mounted on an array of permanent magnets. To provide a smooth magnetic gradient across the microchannel, spacers were introduced to the system adjusting the magnetic field strength. Since blood sedimentation affects capture efficiency, throughput and purity of the system, the array of magnets was arranged with opposite polarities next to each other to introduce a flip–flop motion and thus prevent sedimentation. The cancerous cells were labeled with anti-EpCAM functionalized nanoparticles and passed through the channel. The tagged cells were captured on the substrate and then the coverslip was detached from PDMS for further analysis such as staining for anti-CD45 and anti-cytokeratin. The system was tested for assessing capture efficiency with blood sample spiked with 100,000 cancer cells. The average capture rates were 97%, 107% and 97% for SkBr3, PC3 and Colo205 cells, respectively. In another research, Chen in collaboration with Haung did some modification on the previous proposed geometry [14]. They again utilized the idea of using spacers. The spacers were placed at greater number in the front end of microchannel to reduce magnetic force near inlet, prevent nanoparticle aggregation and therefore eliminate aggregate interference with the identification process since the CTCs were no longer invisible buried ore. The direct contact between magnet and microchannel then retained at the rear end to capture the cells which escape the weaker magnetic field near the inlet. There, in addition to existing permanent magnet, micro-magnet was also introduced to the system to enhance the magnetic interaction between cells and microchannel. The micro-magnets were deposited on the coverslip by implementing inkjet printing. To evaporate the printing ink liquid, the glass slide was moved to hot plate after printing. PECVD was also used to coat the entire structure to protect the patterns against blood flow. Two magnetic flux sources, namely, the external magnetic field generated by permanent magnet and internal magnetic field generated by inkjet-printed micro-magnet, exist in the system. The combination of two magnetic flux sources enhanced the capture rate by 26% compared with using plain glass slide as the substrate. Esmailsabzali et al. [15] also did some preclinical study to examine the feasibility of CTCs separation by immunomagnetic labeling of prostate cancer cells. Unlike the previous studies, the blood-borne prostate cancer cells were tagged by using proprietary monoclonal antibody targeting PSMA instead of EpCAM. The magnetic components of the device were fabricated through mask electroplating process. The channel itself was fabricated via SU8 photo patterning and wet etching. Then, the capture efficiency of the device for isolation was assessed by testing the device using spiked blood samples. The device showed at least 85% capture efficiency in all experiments, confirming the device as a viable option for CTC separation. Shamloo et al. [16] simulated a portable lab on a CD microfluidic device for immunomagnetic separation of tagged target cells. To propel liquid within channel, centrifugal force was suggested. The separation was achieved by employing magnetic force. Their model reveals good compatibility with experimental data. These studies imply the feasibility of utilizing microfluidic platform for cancer research

Here, we propose an integrated microfluidic platform for CTC separation and genetic material extraction. To attain prime separation performance, we modified our primary geometry including introducing deflecting flow as well as proposing diverging geometry. We also conducted a parameter study to assess optimal horizontal position for magnet which decreases the particle dispersion at outlet. In mixing unit, the effect of frequency and voltage on mixing index was evaluated and MI trends with abovementioned parameters was utilized to assess ideal value of voltage and frequency for perfect mixing.

## 2. Materials and Methods

### 2.1. Description of Geometry and Function of Each Unit

**Figure 1:**
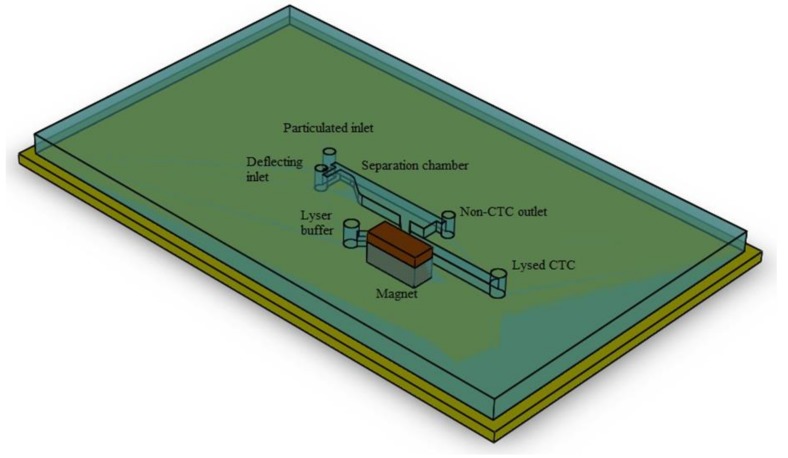
3D schematic of the designed biochip composed of separator and mixer subunits; as can be observed, two units are arranged in serial configuration. In the first chamber, the CTCs are separated from the background cell by implementation of magnetic force and in the mixer unit by utilization of alternating voltage the mixing is achieved.

**Figure 2:**
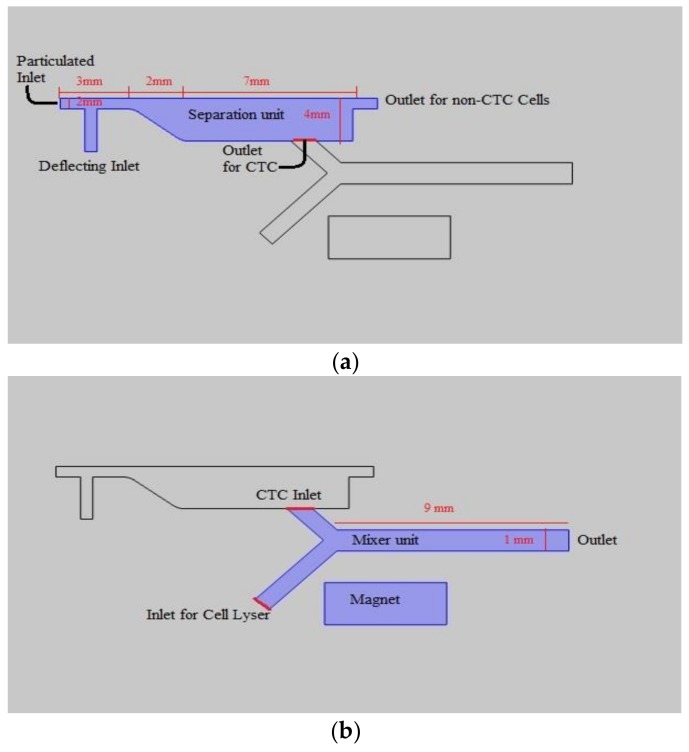
Top view of the geometry: (**a**) Section devised for isolating CTCs, composed of diverging section with 12 mm long in total composed of a 0.5 mm width inlet along with ancillary channel ending up to two distinct outlet one for tagged and one for non-tagged cells; and (**b**) Y-shaped section envisioned as mixing unit.

**Figure 3:**
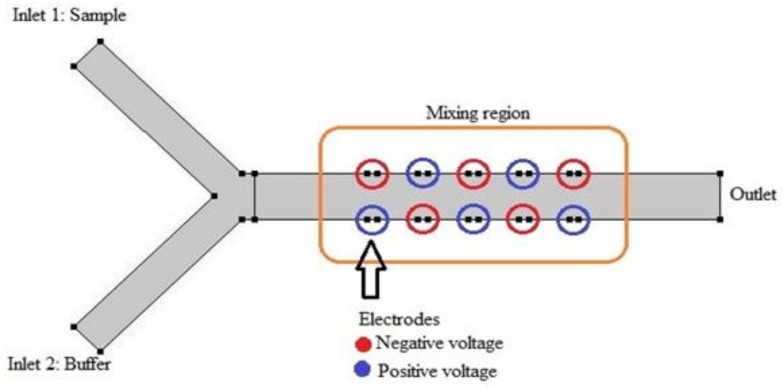
Mixer subunit; the main channel is 9 mm long and is 1 mm wide. As the flow passes the passage, it experiences alternating voltage executed by 10 electrodes which are positioned in zigzag configuration. The slip velocity plays a key role in mixing two parallel streams.

A 3D schematic of the biochip is given in Figure 1. As can be observed, the construct is composed of two subunits arranged in serial configuration. Figure 2 provides a 2D top view of the channel. Figure 2a highlights the portion of the platform which is aimed to isolate CTCs. In this subunit, there exist a main inlet through which the blood sample spiked with the CTCs is introduced. The width of inlet is 500 µm. To amplify the distance between tagged and non-tagged cells at outlet and thus enhance the purity of two streams, this portion is designed to have a diverging profile. Flow enters the inlet channel and moves 3 mm before the commencement of diverging region. There, through a 2 mm long passage, the width of channel reaches 2 mm from 500 µm. The 2 mm width section has a length of 7 mm leading to two outlets. The upper outlet is designated for non-tagged cell departure and has the width of 500 µm, whilst the lower outlet is meant for tagged cell and its width is subsequently assessed by optimizing magnet position. There also exist a perpendicular ancillary inlet through which fresh blood is injected into the chamber. The rationale behind this design is to focus the particles through one channel walls and thus decreasing the size of outlet and resultant pressure loss. The mixer unit is highlighted in Figure 2b. The geometry is rather simple and mixing is not attained by obstacles or curved path, instead electroosmotic phenomenon is employed to achieve mixing. The profile is a Y shaped channel. The length of channel is 9 mm and the width is 1 mm. The CTC-enriched liquid enters from one inlet and the lysis buffer is introduced into the second inlet. Figure 3 provides schematic of this functional subunits. As the two streams flow in parallel, the applied voltage causes slipping the fluid tangent to the wall of channel and induce vortexes which further mix the two flows.

We first simulated each section separately and then ran the model considering real time process within which both subunits work concurrently.

### 2.2. Description of Separation Mechanism

The separation is achieved by immunomagnetic technique. According to the Introduction, CTCs are in fact malfunctioning cells with epithelial root. These cells express specific adhesion molecule (EpCAM) on their surface. The number of these receptors is roughly estimated [17]. The magnetic bead is functionalized with anti-EpCAM antigens. Through antibody–antigen interactions, these beads can be bonded to the surface of circulating tumor cells. The CTCs labeled with magnetic beads then acquire magnetic property which distinguishes them from background cells. The tagged cells can then be manipulated by application of an external magnetic field. In this text the cancer cells are modeled as particles with permeability which differs from the background cells.

### 2.3. Sample Preprocessing

In this study, it is assumed that the blood is preprocessed before running the experiment. Blood is in fact composed of blood cells suspended in plasma. The most abundant cells in blood are RBCs which compose 44% total volume of blood. Existence of these cells within blood makes it a non-Newtonian shear-thinning biofluid. These cells also possess magnetic property which can interfere with those of tagged cells. To reduce the propensity of contamination CTCs effluent with RBCs, it is assumed that the blood is centrifuged in initio and the sedimented RBCs are discarded. The remaining represents a Newtonian fluid and the probability of flow contamination is thus avoided

### 2.4. Separation Unit Governing Equations

#### 2.4.1. Attaining Velocity and Pressure Field

Velocity and pressure field should be attained a priori enabling further analysis. To do so, fluid is assumed to be Newtonian and incompressible and the laminar flow is considered as a consequence non-turbulent model was activated. Both streamline diffusion and crosswind diffusion option were checked to stabilize the solution preventing fluctuations to grow or false diffusion to affect the results. P2 + P1 discretization scheme was selected. Here, the flow field is solved by dividing implementation of the second order elements, whereas the pressure field is attained by application of first order elements. The continuity equation and the momentum equation were solved simultaneously.

(1) $$\rho \nabla \cdot \vec{u}=0,$$

(2) $$\rho \left(\vec{u}\cdot \nabla \right)\vec{u}=\nabla \cdot \left[-pI+\mu \left(\nabla \vec{u}+{\left(\nabla \vec{u}\right)}^{T}\right)\right],$$

#### 2.4.2. Solving for Magnetic Field Distribution

Two major forces act on cells, namely hydrodynamic drag force and magnetic force. Acquisition of drag force require flow field to be determined. In the previous step, the flow field was attained. Assessing magnetophoretic forces constrains the determination of magnetic field distribution in vicinity of the permanent magnet. To do so, Maxwell’s equations are solved. H, M and B can be said to be principle variables in electromagnetic field, where H is magnetic field, M is magnetization and B is magnetic induction. By means of magnetic susceptibility χ, M and H are related such that M=χH (it should be declared if remnant magnetization exists, the aforementioned relation converts into $M=\chi H_{\mathrm{ext}}+M_{\mathrm{rem}}$). Magnetic field governing equations is presented below:

(3) $$\nabla .\vec{B}=0,$$

(4) $${\vec{H}}_{\mathrm{ext}}=-\nabla V_{m},$$

#### 2.4.3. Assessing Particle Deflection and Separation Efficacy

Particle tracing module was implemented to trace the trajectory of tagged and non-tagged cells in the presence of magnetic field. Here, by utilizing Lagrangian description, motion of each particle during time frames is assessed using Newton’s law of motion. Newton’s law of motion requires specification of the particle mass, and all the forces acting on the particle. The forces acting on particles can be divided into two categories, those due to external fields and due to interactions between particles. For each particle, an ordinary differential equation is solved for each component of the position vector. This means that two ordinary differential equations are solved for each particle in 2D. At each time step, the forces acting on each particle are queried from the external fields at the current particle position. Here, we assumed sparse flow which means the effect of individual particle motion on either other particles or flow is eliminated. In other words, the one-way coupling is considered. This necessitates the volume fraction of the particles within the continuous phase to be less than 1%. Newton’s second law states that the net force on a particle is equal to its time rate of change of its linear momentum in an inertial reference frame.

(5) $$\frac{d}{dt}\left(m_{p}\vec{v}\right)={\vec{F}}_{D}+{\vec{F}}_{g}+{\vec{F}}_{ext},$$

Since we are working with 2D geometry, the forces due to buoyancy and gravity are neglected and the magnetophoretic force is assumed to be the only force acting on particle beside the drag force. The drag force is calculated upon Schiller–Naumann formulation:

(6) $$F_{D}=\frac{1}{\tau_{p}}m_{p}\left(\vec{u}-\vec{v}\right),$$

(7) τp=2ρpdp23μCDRer,

(8) CD=24Rer(1+0.15Ref0.687),

Formulation upon which magnetophoretic force is evaluated is given below:

(9) $$F_{m}=2\pi r_{p}^{3}\mu_{0}\mu_{r}K\nabla^{2}H,$$

(10) $$K=\frac{\mu_{r,p}-\mu_{r}}{\mu_{r,p}+2\mu_{r}},$$

### 2.5. Governing Equations for Mixing Subunit

Here, three physics are solved in an independent manner. The physics are laminar flow, electric field and transport of diluted species. The physics in each section are solved based on the assumption that each is unaffected by the others. The study is composed of two steps. First the fluid field is attained for steady state condition and then the effect of alternating current is considered by taking time dependent study.

#### 2.5.1. Assessment of Fluid Field Variable

Flow field was attained by solving Navier–Stokes equation for the incompressible flow. Equations (1) and (2) are again solved. The main difference in this section is that an ionic solution containing free ions is introduced at second inlet as lysis buffer. Due to existence of Debye layer, the fluid near the wall does not obey the no slip boundary condition. Instead, there exists a slipping velocity which can be attained by Helmholtz–Smoluchowski relation [18]:

(11) $$u_{T}=-\frac{\varepsilon_{w}\xi_{0}}{\eta }\nabla V,$$

In this equation, V denotes the electric potentia, $\varepsilon_{w}=\varepsilon_{0}\varepsilon_{r}$ represents the fluid’s electric permittivity and $\xi_{0}$ equals the zeta potential at the channel wall.

#### 2.5.2. Solving for Electric Field

As can be comprehended from Equation (11), assessing tangential velocity field on channel walls necessities awareness of electric field within the confined geometry. To build the electric field, 10 electrodes are embedded in an alternating fashion into the channel walls. Figure 3 illustrates the configuration for better comprehension. The electric potential is then obtained by solving following equation:

(12) $$\nabla \cdot \vec{J}=Q_{j},$$

(13) $$\vec{J}=\sigma \vec{E}+{\vec{J}}_{e},$$

(14) $$\vec{E}=-\nabla V,$$

#### 2.5.3. Acquisition of Transport Characteristics within Channel

Convection–diffusion equation is solved to assess the mixing capability of the device. The equation is as follows:

(15) $$\nabla \cdot \left(Dc\right)+\vec{u}\cdot \nabla c=0,$$

No additional transport mechanism is considered. Both streamline and crosswind diffusion are checked to reduce numerical discretization errors. Quadratic elements are considered to increase the accuracy and precision of evaluation.

#### 2.5.4. Evaluation of Mixing Quality

To assess mixing quality indication parameter, M.I is introduced. To calculate this parameter, first, the concentration distribution is plotted. Then, on a specified location near the outlet pixel, intensity is retrieved. The mass average of concentration is calculated and the deviation of concentration from mean value is obtained. Then, this value is divided by mean average to normalize it. M.I is attained by subtracting the calculated value from one [19,20]

(16) $$CoV=\frac{\sqrt{\frac{\sum {\left(c_{i}-c_{avg}\right)}^{2}}{n-1}}}{c_{avg}},$$

(17) M.I=1−CoV,

## 3. Results

### 3.1. Geometry Evolution of the Separation Subunit

**Figure 4:**
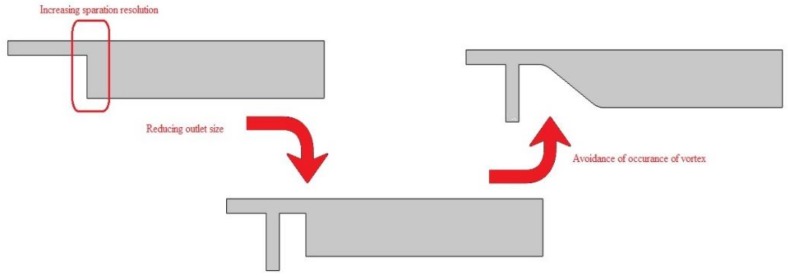
The evolution process of primary geometry: first by introducing an ancillary flow pf particles, dispersion is reduced and then, by gradual increase of cross-section, the induced vortices are avoided.

The geometry of device should be optimized before fabrication to condense fixed asset. A thorough and detailed simulation will reduce the number of experiments in the subsequent step and thereby save time and effort. Here, by exploring particle trajectory, we optimized the position of outlet as well as the magnets position. Some modifications were introduced on the primary geometry. Figure 4 illustrates the evolution process of the initial construct. First, an ancillary inlet channel was added to the geometry and, second, the increase in cross section occurred gradually instead of using a prompt increase in downward chamber.

In designing the separation unit, isolation of the tagged cells with high purity is pursued. This means the CTC enriched stream should not be contaminated with non-tumor cells. The main channel is designed to have a diverging profile to supply the adequate space for tagged and non-tagged particles to depart from each other. The increase in cross section area can occur in two different ways. In one approach, the cross-section can increase promptly and, in another approach, the cross-section could rise gradually. First, we assume prompt increase in cross-sectional region. Since the particles follow the streamlines, for a wide distribution in position of particles at outlet to exist and to for all particles to reach the collecting reservoir, a large outlet is required. A large outlet increases local loss and a higher power should be supplied by syringe pump to propel fluid through the channel. An ancillary perpendicular channel is devised as secondary inlet to focus the particles introduced at main inlet to one side and reduce the deviation in particle positions. Table 1 shows the role of the auxiliary channel in decreasing distance between particles. The simulation was executed by injecting three particles at inlet each of them representative of particles flowing from top, middle, and bottom of the channel. The y position of each particles at channel terminal section was queried in both presence and absence of deflecting flow. As the results indicate the scattering of vertical position lessens by introducing ancillary flow.

**Figure 5:**
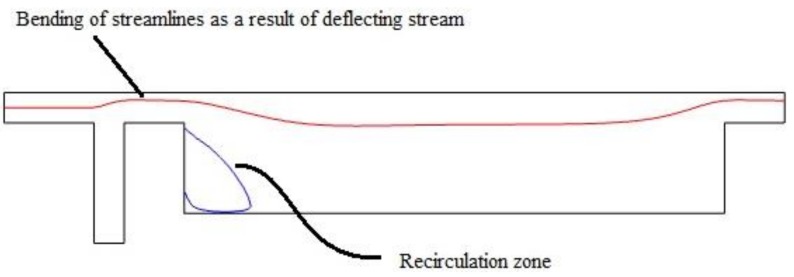
Consequences of adding ancillary channel: from the illustration, it is obvious that, by introducing this perpendicular flow, the streamlines are shifted upward. A circulation zone will also be formed at the bottom edge.

**Figure 6:**
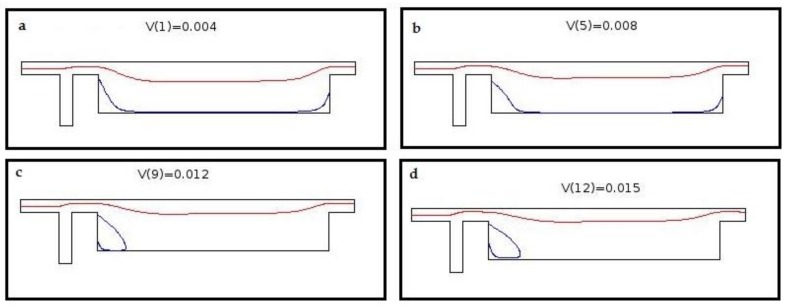
Change in streamlines by increasing velocity of auxiliary channel: (**a**) 4 mm/s; (**b**) 8 mm/s; (**c**) 12 mm/s; and (**d**) 15 mm/s. By increasing the velocity, the streamlines are lifted up and detached from the wall. A circulation zone will be formed above a threshold velocity.

**Figure 7:**
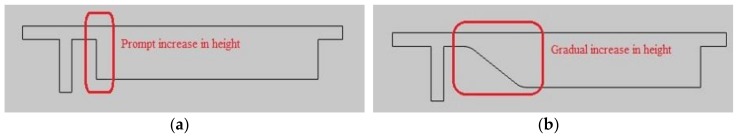
Representation of possible ways for increase in cross section: (**a**) prompt increase; and (**b**) gradual increase.

Introducing this perpendicular flow, despite helping decrease the size of outlet, hosts an adverse effect. This flow pushes the main stream upward so it produces a secondary flow at the corner on main geometry. This circulation zone increases in size as the ancillary flow increases. Figure 5 shows the formed circulation zone for ancillary velocity of 12 mm/s. Figure 6 depict the changes in streamlines arrangement due to increase in auxiliary flow rate. These recirculation zones are undesired since some cells might become trapped in them and the consistency and accuracy of the device will drop as a consequence. Therefore, instead of prompt increase in cross section, we choose a gradual increase in cross section. The two profiles are given in Figure 7.

**Figure 8:**
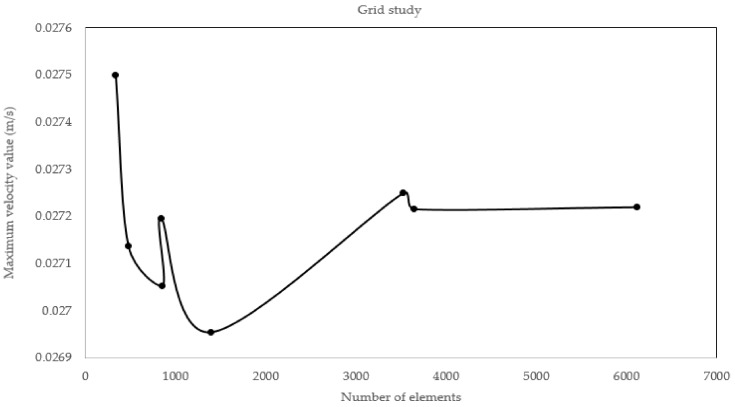
The way by which the domain is discretized has profound effect on accuracy of solution. Increasing number of elements increases both the accuracy and run time. The solution must be independent of element number. As can be observed, if the domain is discretized with more than 3000 elements, the maximum velocity becomes independent of number of elements.

After finding the optimal geometry, we performed a grid study to ensure independence of the solution from mesh. To do so, we gradually increased the number of cells and the maximum velocity was queried. It was observed there exists a threshold above which increasing element number would make no difference in the value assessed instead increasing computational cost. Thus, we choose this limit as appropriate element number. Figure 8 depicts the relation between number of elements and maximum velocity value assessed.

**Figure 9:**
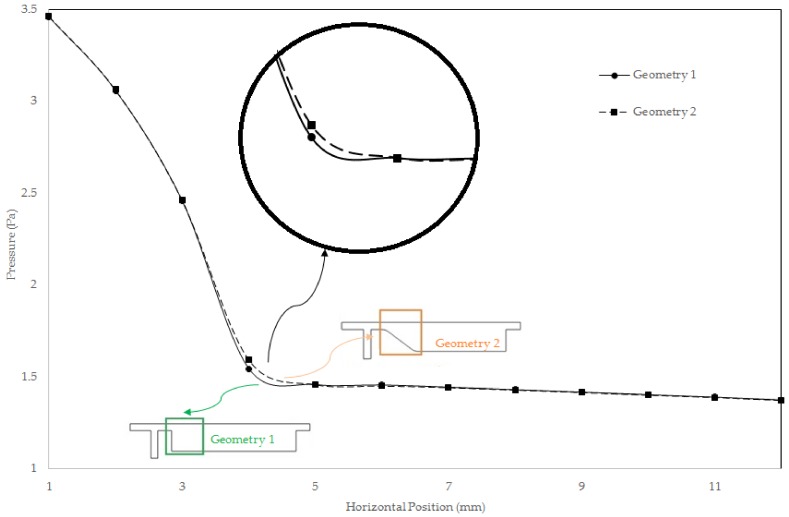
Pressure loss across the channel for two proposed geometry: in both geometries, the pressure decreases stream-wise and for Geometry 1 the loss is greater.

**Figure 10:**
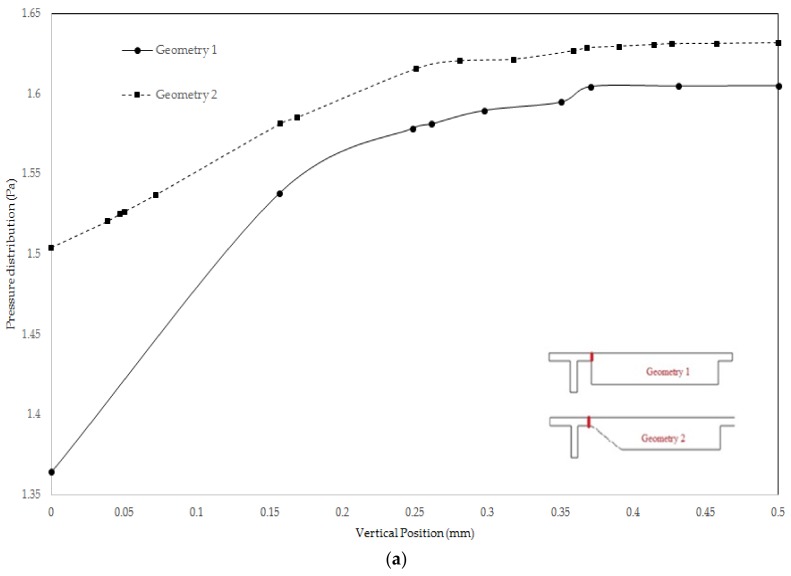

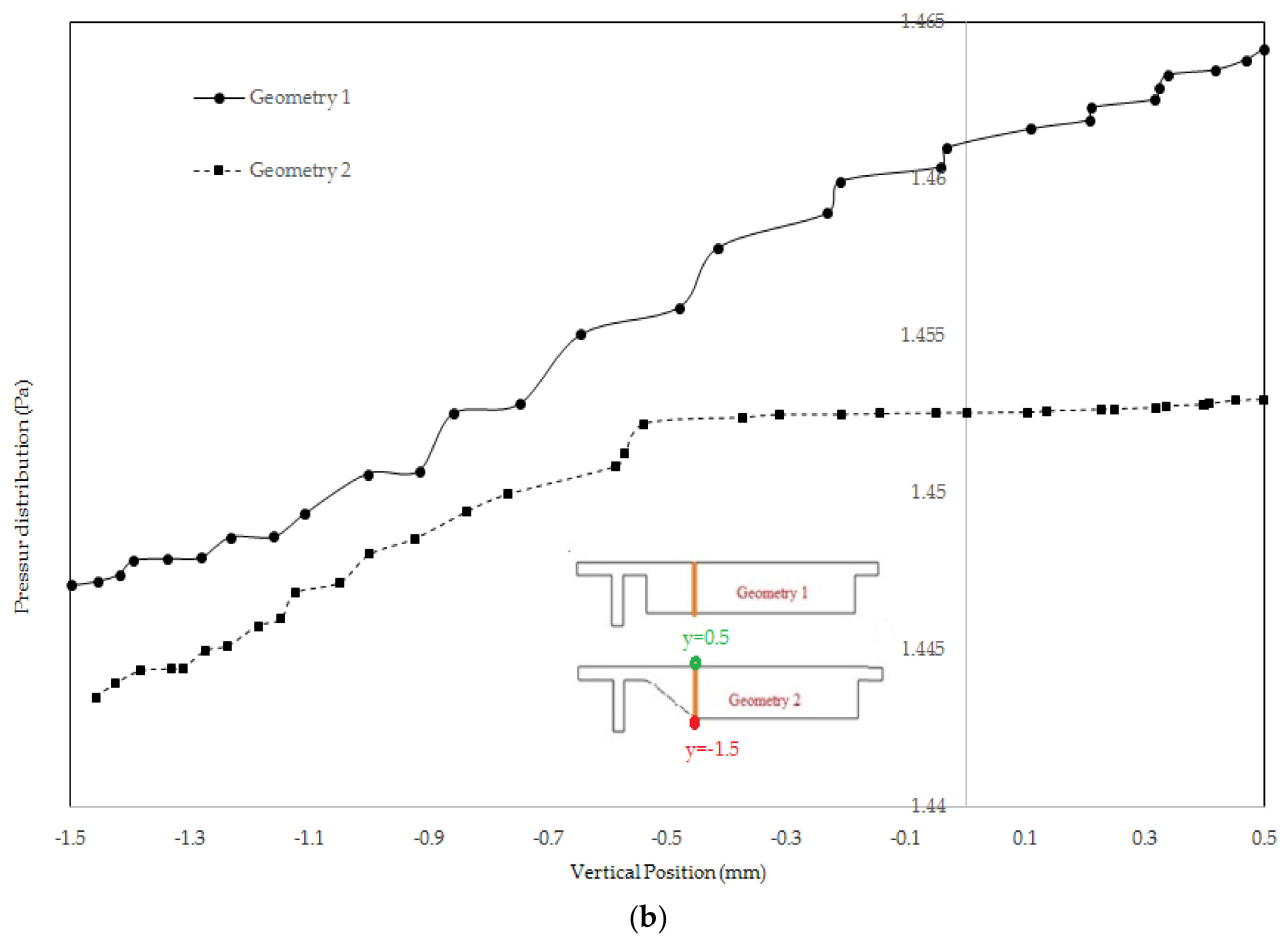
Pressure at specified cross section; the cross sections are determined to be: (**a**) at the beginning of the diverging section; and (**b**) at the immediate end of diverging section.

Following to grid study, we evaluated pressure drop across a line along main channel. The result is given in Figure 9. As can be comprehended, the local pressure loss is less in gradual channel profile. Pressure distribution at inlet of main channel and the immediate end of increase region for two geometries is given in Figure 10a,b. As it can be observed, at Section 1, the pressure is higher in Geometry 2, whereas, at Section 2, the pressure is higher for the geometry one. Thus, the gradual increase in cross section while preventing formation of vortices increase pressure loss due to increase in path the flow travel and frictional loss therefore becomes greater.

### 3.2. Velocity and Magnetic Field within the Confined Geometry

**Figure 11:**
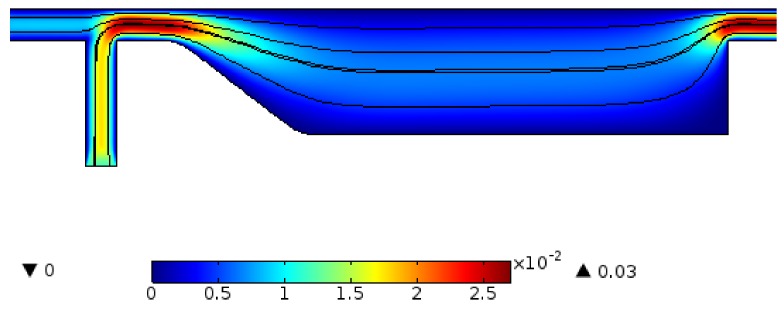
Velocity field within the separator geometry: at the cross section of two inlet flows, the velocity reaches its maximum. The streamlines are also illustrated. For applicability of device for extensive cellular study, one must ensure that the shear rate within the channel does not violate the maximum allowable shear. Otherwise, the cell viability is critically imperiled.

**Figure 12:**
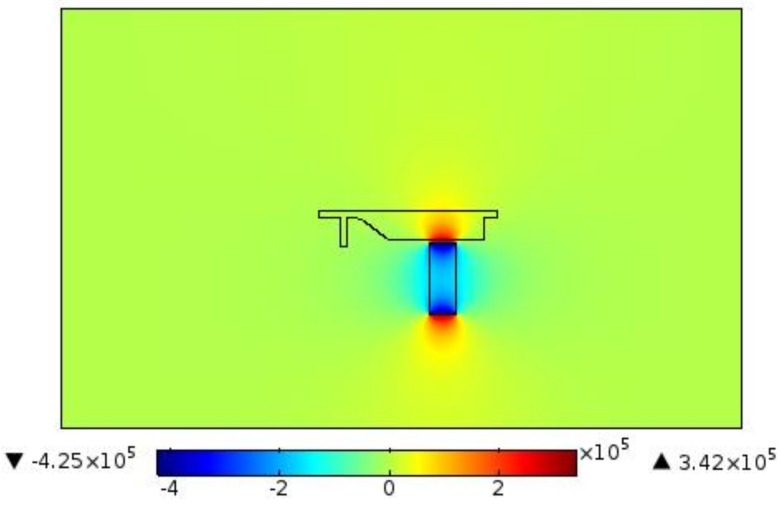
The magnetic distribution: the field maximum is located near the pole and there exists a rapid decay in the magnitude of magnetic field. The magnet only affects a small vicinity around it.

**Figure 13:**
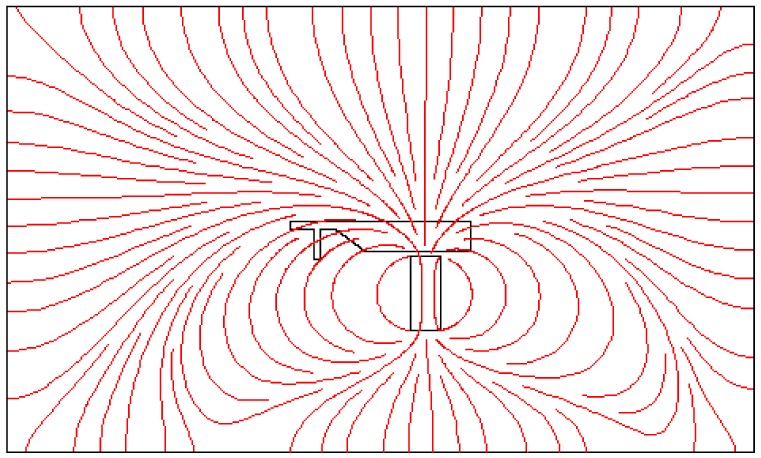
The magnetic field lines: the lines form an asymmetrical pattern with high density near the poles and low densities at other places.

**Figure 14:**
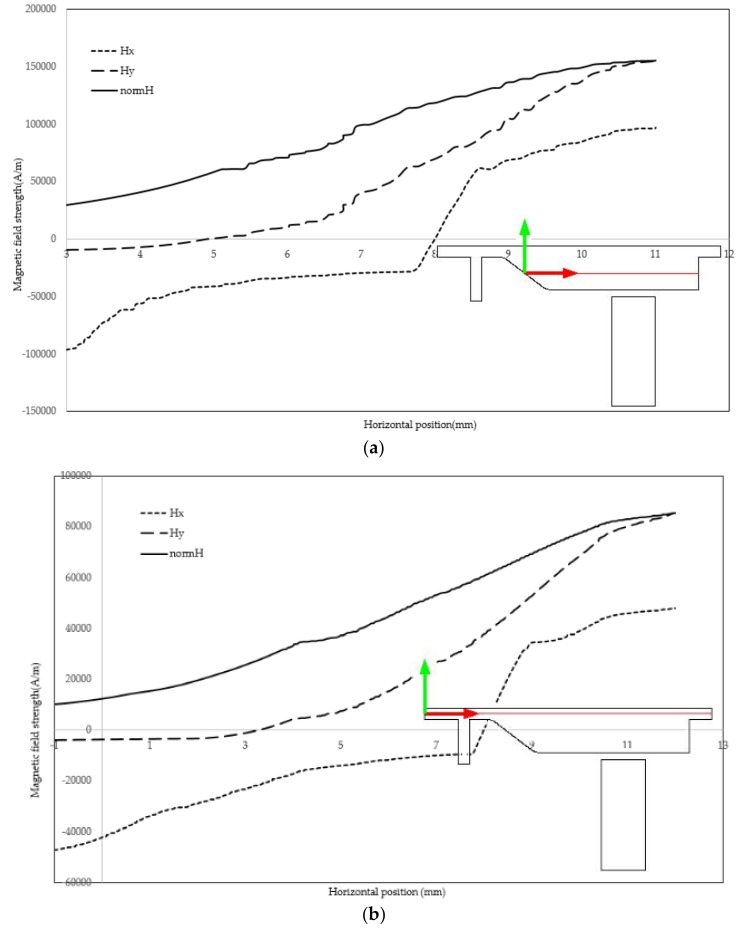
The magnetic field strength: (**a**) field magnitude in vicinity of bottom wall; and (**b**) field magnitude in vicinity of top wall.

Figure 11 illustrates the velocity field within the separator. If the aim is to culture CTCs for further investigation, the viability of cells must be preserved. This requires the maximum shear stress not violating the allowable limit. Numerical simulation of flow within channel enables estimating flow related variables and tuning them within desired ranges. The magnetic field is shown in Figure 12. The maximum field is near the pole and there exists a rapid decay in the magnitude of magnetic field. The magnet was simulated by defining magnet domain as magnetic flux conversion node having magnetization of 750 kA/m. The maximum magnetic flux density was obtained to be 1.05 T which is comparable to magnetic flux density of conventional magnet. The magnetic field lines are given in Figure 13. The lines are asymmetrical with high density near the poles and low densities at other places. In addition, magnetic field strength components H_x_ and H_y_ across the channel as well as magnetic strength at two positions are given in Figure 14a,b.

### 3.3. Attaining the Optimized Position for Magnet

**Figure 15:**
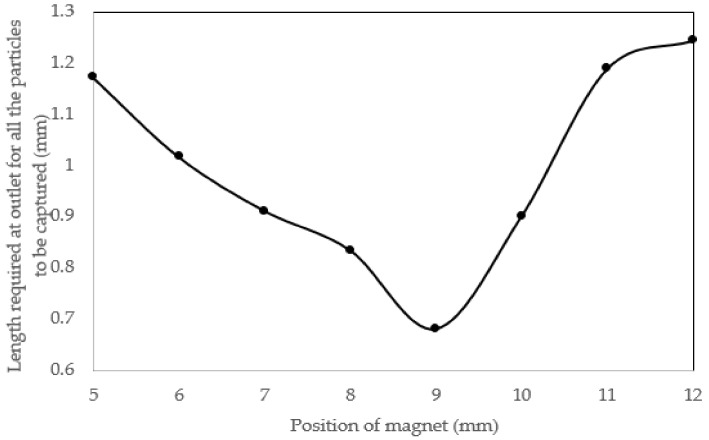
The position of magnet versus the required size of outlet: as the magnet moves in horizontal position far away from inlet particle, dispersion decreases and reaches a minimum value above this extreme point further moving of magnet increases the distance between particles hitting the bottom wall.

The position of permanent magnet has profound effect on magnetic field distribution hence the force acting on the tagged particles. We conducted a parametric study in which we displace the magnet within a specified range and assess the minimum and maximum horizontal position through which the particles hit the bottom wall. The simulation indicated there exists an optimum position for magnet where the deviation in particles position is minimized. It can be seen that, by moving magnet in horizontal manner towards the channel outlet of channel, the distance between the particles reaches a minimum value, which is favored. Reduction in outlet size would similarly focus the flow, which is sought for separation purposes. By this means, the output stream can be more precisely controlled. Additionally, increasing the size of the tagged cell exit would eventually decrease the flow resistance and thus more flow is drawn to this channel. The flow pattern is therefore affected and the unwanted contamination is probably increased. In addition, the larger is the outlet, the greater embedded volume will be and the power required for flow propulsion will increase, which seems unnecessary. Further displacement of the magnet again increases the distance between particles. Figure 15 shows this trend. The numerical values are also given in Table 2.

### 3.4. Quantification of Device as a Separation Unit

**Figure 16:**
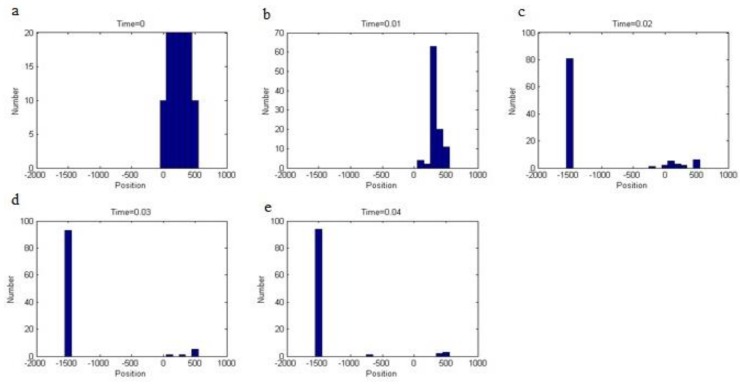
The histogram of particle vertical position at different times: the position is given in microns. As the time progressed (moving from graph (**a**–**e**)), the band width of particle distribution is reduced and the particles are all gathered in the same location.

**Figure 17:**
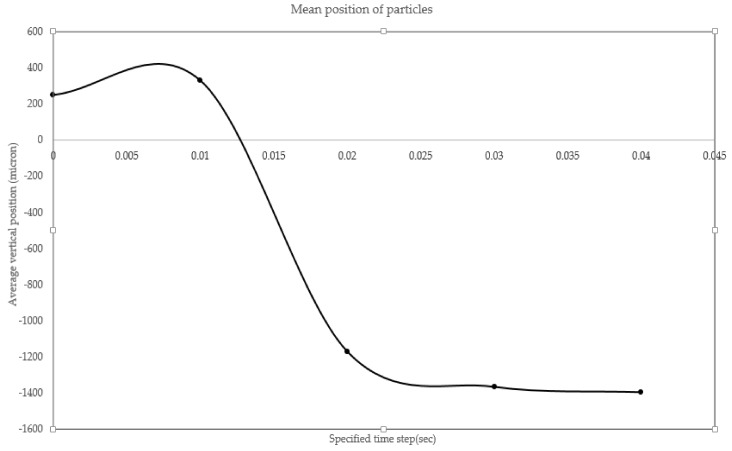
The mean position of injected particle versus time: the particles experience a steep change during 0.01–0.02 and then the change in position varies with a mild slope.

It is pursued to separate particles with high throughput and minimal contamination by non-tagged cells. To quantify the effectiveness of the separating unit, we inject 100 particles at inlet and trace their trajectory thru different time frames. We first queried the vertical position of all 100 particles at time t = 0, 0.01, 0.02, 0.03, and 0.04 s. Then, we obtained the histogram of particle distribution in different location. These histograms are presented in Figure 16. At the beginning, there exists an even distribution of particles throughout the channel width. Over time, the particle distribution shifts rapidly towards the channel bottom. As another step in quantifying the separator unit, the mean position of particles is evaluated at previously defined time steps. At given times, individual particles are assessed, and then they are averaged upon the whole number of particles. The mean position of particle is given in Figure 17. Both the beginning and end particles revealed a slightly changed vertical position, whereas, at the intermediate times, steep change in vertical position is observed.

### 3.5. Order of Magnitude Analysis to Figure out Dominant Forces Exerting on the Particles

Particle transport in microchannel under external magnetic fields is governed by various forces and interactions. This includes the magnetophoretic force; drag force exerted on particles due to relative motion between particle; and fluid, gravity, and buoyancy forces. Particles also interact with microchannel surface. They also exhibit Brownian motion due to thermal fluctuation. There also exist particle–fluid interaction and interparticle effects. For the case studied here, only the magnetic and viscous forces are dominant. Below, we make a valuation which gives us an insight of order of magnitude of engaged forces, which further help us to decide which forces to consider. We start with gravitational and buoyant forces. The gravitational force is obtained by $F_{g}=\left(\frac{4}{3}\pi R_{c}{}^{3}\right)\rho_{c}g$ and the buoyancy force is attained by $F_{b}=\left(\frac{4}{3}\pi R_{c}{}^{3}\right)\rho_{f}g$. For a 10 µm cell (ρ_c_ = 1050 kg/m^3^, ρ_f_ = 1000 kg/m^3^), we obtain F_g_ = 5.13 pN and F_b_ = 5.3 pN, both of which are two orders of magnitude smaller than magnetic buoyancy force. The molecular and electrical interaction between particles and surfaces of microchannel is referred as Derjaguin–Landau–Verwey–Overbeek (DLVO) force. DLVO force is a combination of van der Waal’s force and electrostatic force. Electrostatic force can be either repulsive or attractive, while van der Waal’s force is always attractive. It is usually beneficial to treat surface of microchannel chemically and render the electrostatic force repulsive to avoid problems of particles sticking to the channel surface. DLVO force can be neglected after proper surface treatment. We assume that the specimen is diluted in such way that the volume fraction of the particles within the continuous phase is less than 1% so interparticle forces can be neglected.

### 3.6. Grid Study for the Mixer

**Figure 18:**
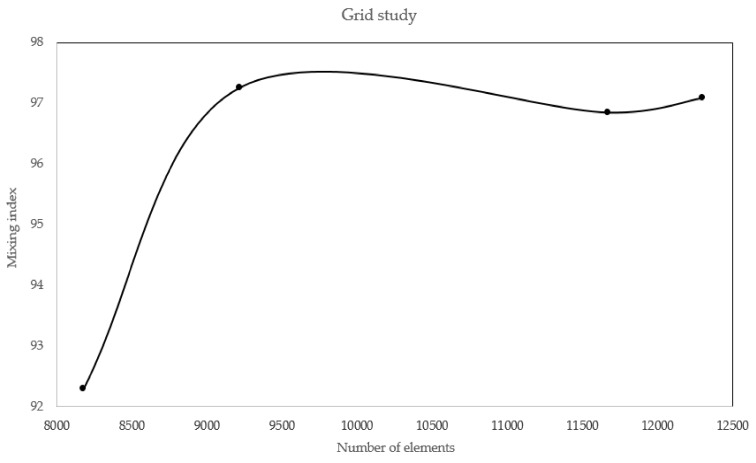
The accuracy of numerical solution is tightly bound to the grid. Increasing number of element increases both the accuracy and run time. The solution must be independent of element number. If the domain is discretized with more than 11,000 elements, the mixing index becomes independent of number of elements.

To guarantee that the solution is independent of grid, the mesh dependency was studied. This step plays a key role in ensuring the consistency of solution. To perform a grid study, the solution was carried out several times with increasing the number of elements at each step. After each evaluation, an important field variable was calculated and the trend of variable with number of elements was depicted. To ensure the grid dependence, this graph must yield smooth non-varying variable with different element number. We first discretized the domain with 8000 elements and then gradually increased the element number to 12,000 in multiple steps. At each step, mixing index is assessed, and then the obtained values were plotted versus element number. The desired profile can be viewed in Figure 18, ensuring the grid independency.

### 3.7. Effect of Voltage and Frequency on Mixing Index

**Figure 19:**
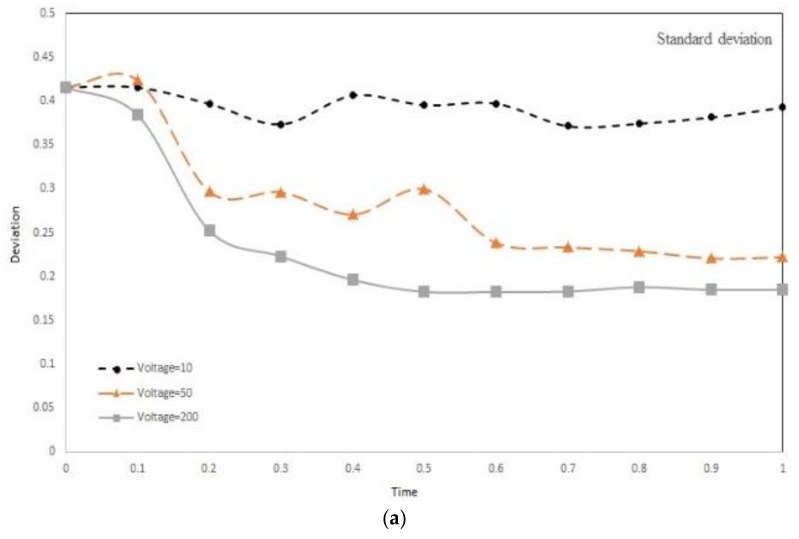

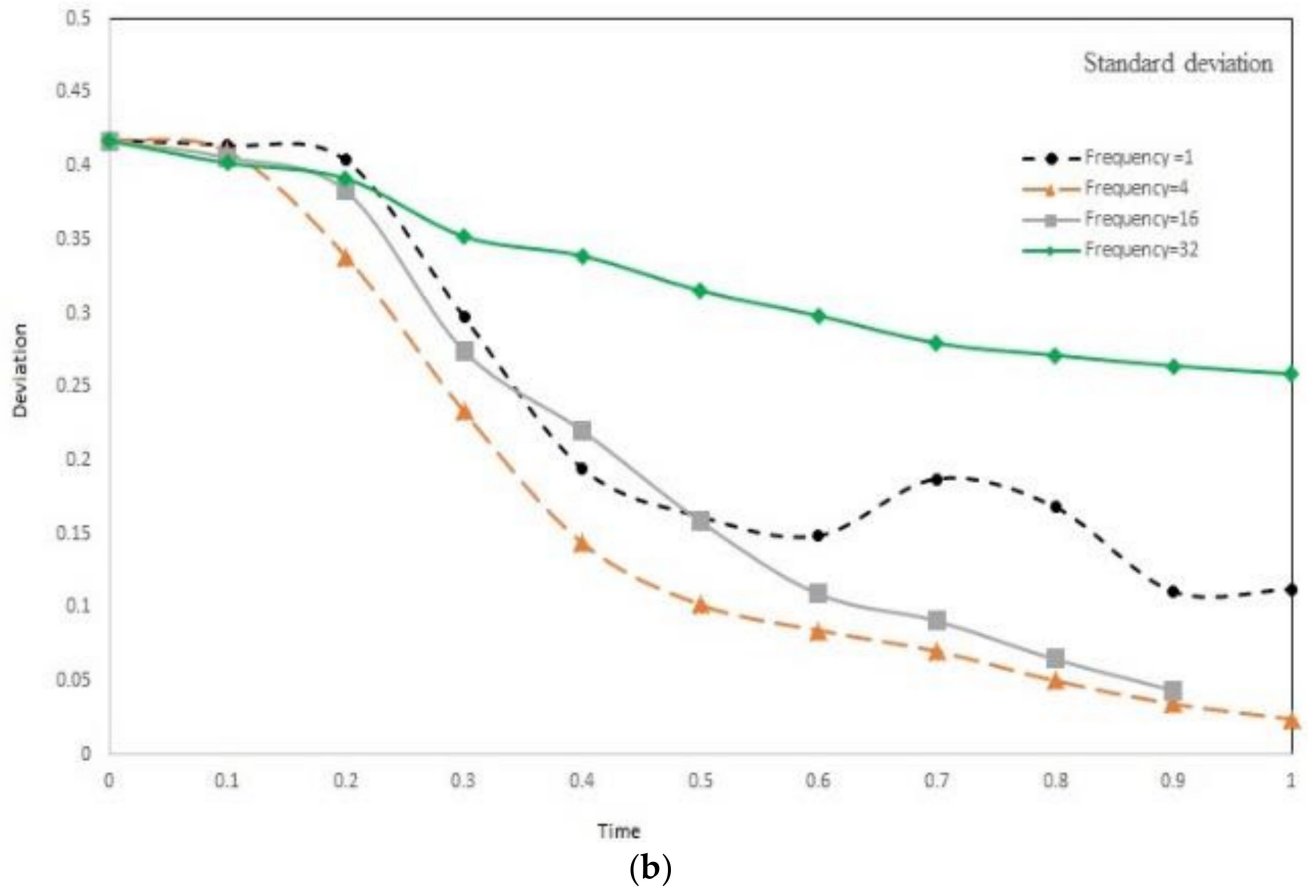
Assessing quality of mixing: (**a**) Deviation in pixel intensities at outlet versus time at different voltages: by increasing voltage the deviation is reduced. (**b**) Deviation in pixel intensities at outlet versus time at different frequencies: Mixing first improves as the frequency rises but above a certain frequency the performance deteriorates by further increase in frequency.

The ultimate goal of a mixer is to yield a high mixing index. Mixing index is affected by many parameters based on the technique utilized. In Electroosmotic mixing, applied voltage and the frequency play a key role in the resultant MI. Here, by employing transport of diluted species and the electric field module, we studied the effect of above-mentioned variables on mixing efficiency. Three physics should be solved concurrently. The flow field should be attained since, in evaluating mixing, we must solve transport of species equation and this requires the velocity to be known since the convection term embed the velocity in it. The velocity tangent to wall is due to Debye layer and presence of electrodes, thereby the electric field should be solved. We performed two independent studies. In one study, we kept all other involving parameters constant and the voltages 10 V, 50 V, and 100 V are supplied to the electrodes. As can be observed in Figure 19a, by increasing the voltage, the deviation in pixel intensities is reduced and thus the MI is enhanced. This indicates that increasing voltage has positive effect on mixing efficiency of the device. This finding is consistent with what we expected. In the Electroosmotic mixing, mixing is achieved by slipping the fluid adjacent to the wall due to Debye layer effect. This tangential velocity, as presented in Equation (11), is directly proportional to the gradient of the electric field. By increasing the applied voltage, the electric field is magnified and the mixing is improved. The main hurdle is that, by increasing voltage, we also amplify the resistive loss and therefore the temperature in the device may rise above biological allowable range rendering environment damage to the cells and biological material. The effect of frequency on MI was also studied. The studied frequencies were 2 Hz, 4 Hz, 16 Hz, and 32 Hz. Unlike voltage, the frequency exhibit varying behavior. Increasing frequency first improves mixing, and then above a critical frequency, further increase deteriorates MI. the underlying reason is that above the critical frequency the system cannot follow the changes since its response time is greater than the change duration. Thus, an optimal frequency exists for yielding maximum MI. Here, the frequency of 4 Hz is shown to be the optimal frequency for mixing. The response time of the system is below 4 Hz and, above this value, the system the rate of change is so quick, the system cannot perceive the changes and therefore the mixing efficiency worsens.

## 4. Discussion

Early diagnosis of cancer and assessing tumor biology hold great importance in treating cancers. Here, we simulated the microfluidic biochip envisioned for CTC isolation and genetic material extraction. The device is composed of a separation unit and a mixing unit organized in a serial configuration. The primary geometry while being simple yielded low purity and separation efficiency. To overcome the shortcomings, such as contamination of CTC enriched stream with non-tagged cells, some modifications were proposed. Figure 4 depicts the evolution of separator geometry. In the first step, a perpendicular channel was considered. The rationale behind this was to pinch the particulated flow, thus reducing the dispersion length of particles and consequently decreasing the size of outlet. It was found that the velocity of deflecting flow has profound effect on dispersion length. The larger is the velocity, the smaller the dispersion length will be. Large velocities also result in flow detachment from the wall formation of circulatory zone. Figure 6 illustrates this matter in detail. Vortex are undesired since they cause energy loss and some particles might become trapped within them. Considering this, in the next step, instead of prompt increase in cross section, it was decided to enlarge the section gradually. With gradual section increase, the sharp corner edge was eliminated and consequently the vortex formation is prevented. For the two geometries, flow field studies were conducted as well. The results are illustrated in Figure 9 and Figure 10. Figure 9 shows the change in pressure across channel length. The pressure first decreases rapidly and then reduces with a mild slope. The local pressure loss in Geometry 1 (with prompt cross-section increase) is greater but nearly at the immediate end of diverging section pressure in both geometries approaches to same curve. In Figure 9, the two geometries are similar in their flow field characteristics. Apart from similar fluidic characteristic, Geometry 2 also prevents the circulation zone formation. Figure 10, in contrast, considers the pressure variation in two cross sections: one in the beginning of the diverging region and the other at its immediate end. Pressure near the bottom wall is more grated than pressure in vicinity of the top wall. To attain flow field, both continuity and conservation of momentum equations were solved concurrently and the velocity magnitude in different elements was calculated. Figure 11 represents the calculated flow field. For flow field study, the maximum velocity in the domain should be assessed to ensure that laminar flow assumption was not trespassed. If the viability of cells is desired, the maximum shear stress should also be queried to ensure that it does not violate the allowable shear stress threshold. Here, we utilize magnetophoretic cell separation. To this end, the CTC cells are labeled via magnetic nanoparticle. In the presence of external magnetic field, the tagged cells depart from main stream and are guided towards the desired outlet. Prior to evaluation of magnetic force, the magnetic field distribution should be attained. Figure 12 depicts the magnetic field strength. The maximum value is detected near the pole and the field magnitude rapidly decreases by going far away the magnet. The magnetic field lines are given in Figure 13. The lines are more focused near the poles and they diverge as the go further. The field strength consequently decreases to great extent far from the magnet. The position of magnet was also optimized. To do so, through several steps, the magnet was displaced and position at which the particles hit the bottom wall was attained. Figure 15 shows the trend in dispersion length. As can be observed, there exists an optimal position by which this distance is minimized. The magnet is placed in this position and remaining simulation was carried out by considering the magnet there. The grid study was also conducted and the independence of solution from mesh was guaranteed. To assess mixing index laminar flow, transport of diluted species and electrical field were solved simultaneously. The concentration plots were produced and by an in-house code the MI was evaluated. We first studied the effect of applied voltage on mixing index and discovered that, by increasing applied voltage, the quality of mixing is enhanced. However, there exists biological constraints and the voltage cannot be increased unboundedly. Exploiting higher voltages will in turn increase resistive loss and lead to temperature elevation within the system. This temperature rise might render the environment harsh for cells and compromise their viability. The same study was conducted to investigate the effect of frequency on mixing index. The frequency increased from 2 Hz to 32 Hz. It was observed increasing frequency first improves mixing and then, above a critical frequency, further increase deteriorates MI.

The study was carried out by neglecting particles interaction. It is suggested to consider interparticle forces for more realistic simulation. To reduce the number of elements and therefore computations time. We simulated a 2D construct. It is more accurate to simulate biochip in 3D space. In the calculation of drag force, Schiller–Naumann formulation was employed. This formulation is only consistent when a particle is moving in an infinite fluid. Here, the size of particle is comparable to channel dimension and the effect of wall should also be considered.

## 5. Conclusions

Due to significance of CTCs in therapy monitoring, we propose a microfluidic construct which enables the study of biological material of the circulating tumor cell. The biochip is composed of two functional subunits, namely separating unit and mixing unit. The working principle of the device is simple. In the first step, CTCs are separated from the blood specimen and, in the second step, the CTC enriched stream is mixed with lysis buffer to extract the biological material of the cell. The separation unit and mixing unit both utilize the external forces, namely magnetic and electric forces. The separation is achieved by immunomagnetic technique. In this technique, a functionalized magnetic bead is bonded to the surface of the CTC via antibody–antigen interaction. The cell then can be manipulated with magnetic field. The geometry of the unit is devised to yield a high purity and minimize the probability of the contamination of the tagged cell stream with non-tagged cells. To assess particle trajectory, Newton’s second law of motion was solved for each particle. We did order of magnitude study which gave us an insight of significance of engaged forces. This further helped us disregard trivial forces. To reduce the computation time and for the sake of simplicity we neglected interparticle forces and consider one-way coupling. The position of the magnet was also optimized by doing parametric study. In this section, the goal was to find the horizontal position of magnet which minimizes the dispersion of tagged cells at outlet. The CTC enriched stream is then guided through the mixing region where the flow is agitated by means of an alternating current. The underlying philosophy of this section was to introduce an ionic solution as lysis buffer and exploit Helmholtz–Smoluchowski relation to find slipping velocity adjacent to channel wall. The sliding of the fluid near the wall agitates the flow and result in mixing. Here, parameters such as applied voltage and frequency play key roles in the mixing efficiency. The effect of applied voltage and frequency on mixing index is studied to find the optimal voltage and frequency which provides better mixing. To ensure the independence of results from the grid, a mesh study was done. At the final stage, we simulated the integrated biochip within which two functional subunits worked concurrently. These studies, which were carried out on the construct, reveals its potential for diagnostic application. In future studies, we aim to enhance the performance of platform by experimental implementation of ideas presented in this study and our previous studies [19,20,21,22].

## Figures and Tables

**Table 1:** The minimum required length at upper outlet for all particles to be captured: the data consider both configurations, namely in absence and presence of ancillary channel. Including a perpendicular channel reduces the length required.

**Without Deflecting Channel**				
Vertical Position of Particles (mm)			Min.	−0.98364 mm
Particle 1	Particle 2	Particle 3	Max.	−0.00295 mm
−0.00295	−0.4913	0.98364	Diff.	0.98069 mm
**Without Deflecting Channel**				
Vertical Position of Particles (mm)			Min.	−0.16094 mm
Particle 1	Particle 2	Particle 3	Max.	0.22983 mm
0.22983	0.003208	−0.16094	Diff.	0.39077 mm

**Table 2:** This table contains magnet location and the position of particles representative of particles flowing the top middle and bottom of inlet channel; as can be observed, there exists a point at which the distance between particles is minimized.

Position of Magnet (mm)	Horizontal Position of Particle (mm)			Dispersion Distance (mm)
	Particle 1	Particle 2	Particle 3	
X = 5	5.6101	5.8540	4.6818	1.1722
X = 6	5.9842	6.4064	5.3900	1.0164
X = 7	6.1083	7.0192	6.1233	0.9109
X = 8	7.7192	6.8852	6.9711	0.8340
X = 9	7.3598	8.040	7.8558	0.6802
X = 10	8.1068	7.8119	8.7126	0.9007
X = 11	8.3495	9.0202	9.5384	1.1889
X = 12	9.1278	10.372	10.328	1.2442

## Abbreviations

CTC	Circulating Tumor Cells
FACS	Fluorescence-Activated Cell Sorting
MACS	Magnetic-Activated Cell Sorting
RBCs	Red Blood Cells
